# Carbonaceous Materials Coated Carbon Fibre Reinforced Polymer Matrix Composites

**DOI:** 10.3390/polym13162771

**Published:** 2021-08-18

**Authors:** Bidita Salahuddin, Shaikh N. Faisal, Tajwar A. Baigh, Mohammed N. Alghamdi, Mohammad S. Islam, Bing Song, Xi Zhang, Shuai Gao, Shazed Aziz

**Affiliations:** 1 ARC Centre of Excellence for Electromaterials Science, Intelligent Polymer Research Institute, University of Wollongong, Innovation Campus, Squires Way, North Wollongong, NSW 2522, Australia; shaikh.faisal@uts.edu.au; 2 School of Electrical and Data Engineering, University of Technology Sydney, Sydney, NSW 2007, Australia; 3 Department of Mechanical and Production Engineering, Islamic University of Technology, Gazipur 1704, Bangladesh; tajwar.azim@gmail.com; 4 Department of Mechanical Engineering Technology, Yanbu Industrial College, Yanbu Al-Sinaiyah City 41912, Saudi Arabia; alghamdim@rcyci.edu.sa; 5 School of Mechanical and Manufacturing Engineering, University of New South Wales, Sydney, NSW 2052, Australia; m.s.islam@unsw.edu.au; 6 Scion, Te Papa Tipu Innovation Park, 49 Sala Street, Private Bag 3020, Rotorua 3046, New Zealand; bing.song@scionresearch.com; 7 School of Chemical Engineering, The University of Queensland, St. Lucia, QLD 4072, Australia; xi.zhang4@uq.net.au (X.Z.); s.gao@uq.edu.au (S.G.)

**Keywords:** polymer composites, carbon fibre, carbon nanotube coating, graphene coating, chemical vapor deposition, fibre/matrix interface, mechanical properties

## Abstract

Carbon fibre reinforced polymer composites have high mechanical properties that make them exemplary engineered materials to carry loads and stresses. Coupling fibre and matrix together require good understanding of not only fibre morphology but also matrix rheology. One way of having a strongly coupled fibre and matrix interface is to size the reinforcing fibres by means of micro- or nanocarbon materials coating on the fibre surface. Common coating materials used are carbon nanotubes and nanofibres and graphene, and more recently carbon black (colloidal particles of virtually pure elemental carbon) and graphite. There are several chemical, thermal, and electrochemical processes that are used for coating the carbonous materials onto a carbon fibre surface. Sizing of fibres provides higher interfacial adhesion between fibre and matrix and allows better fibre wetting by the surrounded matrix material. This review paper goes over numerous techniques that are used for engineering the interface between both fibre and matrix systems, which is eventually the key to better mechanical properties of the composite systems.

## 1. Introduction

Carbon fibre (CF) reinforced polymer composites have been consistent in gaining high importance in the area of composite science and technology [1,2,3], particularly in aerospace [4], defence [5], sports goods [6], and automotive industries [7]. The combination of polymer matrix and reinforcing fibres results in high-performance materials that offer weight reduction of more than 50% compared to those of aluminium and steel, respectively [3]. CF reinforcement commands the strength and modulus properties of CF reinforced polymer composites and is the primary load element. The composite material performance usually depends on the interfacial characteristics of the reinforcement and matrix material [8]. It is imperative to have a high-volume fraction of fibres (around 55–60%) to obtain high mechanical performance [9]. Nevertheless, the low interfacial strength and the shortage of interfacial covalent bonds are caused by the even and unreactive characteristics of the CF surface, thereby affecting the mechanical properties of composites [10,11]. It is a long, prevailing, and highly important issue needed to address for the continuous progress of CF reinforced polymer composites in prospective advanced applications. Several research works have been focusing on examining and comprehending the physicochemical interactivity at the fibre/matrix interface [12,13,14,15,16]. The sufficient amount of hydrogen bond and Van der Waals forces between the CF and matrix are essential throughout composite processing for strong interfacial adhesion. The fibre/matrix interfacial adhesion energy should be higher than the cohesive energy of the matrix [17,18,19,20]. The high-performance polymeric composites’ mechanical properties can be improved by modifying CF surface and structure [21,22]. Still, the importance of controlling the fibre/matrix interfacial characteristics is a key task. CF has a non-polar surface with the structure of crystallized graphitic basal planes. The chemical inertness of the CF is caused by the high-temperature carbonization or graphitization during manufacturing [23]. In addition, inadequate bonding with the matrix materials during the manufacturing is triggered by the surface lipophobicity, extreme smoothness and low adsorption characteristics of CF [24]. Thus, strong fibre/matrix interfacial adhesion for successful transfer of stress at the interface is executed by the modifications at the fibre surface to resolve the inertness of CF hierarchically reinforced composite structures [25,26,27,28,29,30]. Significant advancements have been attained in CF reinforced composites for the fibre/matrix interfacial strength and matrix governed by the thickness performance, for example, fracture toughness, fatigue life, impact strength, and interfacial shear strength on the basis of their distinctive structures and modulus and outstanding strength [31,32,33].

Sizing technology has been recently appraised for the modification of CF surface through micro- or nanocarbon materials coating and improvement of fibre/matrix interfacial adhesion [34]. In regard to sizing, in the presence of fibres, retardation of the main crack and an increase in fracture work because of the debonding of fibre and matrix pull out can be possible due to the dissipation mechanism of main energy under mechanical load [35]. It is important to ensure good adhesion of coating onto fibre (in this limiting case; chemical bonding) for resisting the fibre oxidation, otherwise the chance of fibre getting deboned and pull out from the matrix will be higher [15,36,37,38,39,40].

The technologies of fibre coating were established a long time ago for numerous purposes. Carbon nanomaterials such as carbon nanotubes (CNTs), carbon nanofibres (CNFs), graphene, carbon black, and graphite have been popularly employed [41,42,43,44,45,46,47,48,49]. Of them, CNT and graphene have increased noteworthy developments in the electrical characteristics, tensile strength, and barrier performance of fibre [50,51]. CNTs are the precursor in this regard, due to their extraordinary inherent properties; for example, mechanical, physical, electrical, thermal, opto-electronical, and field emission [52,53,54,55,56]. The complications related to the matrix predominating properties can be minimized by the advanced fibre reinforced polymer composites materials through this coating technique. The utilization of CNT in the large volume of polymer composites is an established technique and is utilized well by researchers in-depth [57,58,59,60,61,62,63,64]. Depending on the types of precursor coating materials, several coating techniques have been proposed, including chemical vapour deposition (CVD) [65,66,67,68,69,70,71], dip coating [70,72], electrophoretic deposition [73,74,75], and spray coating [76]. The highly crystallized graphite basal plane sites can be functionalized by these methods and raises their surface energy. Due to the addition of reactive functional groups or pitting, the fibre surface becomes roughened. As a result, mechanical interlocking between fibre and polymer, which imparts interfacial strength to the composite materials, is improved [77,78,79]. The largest batch of nanocarbon coated fibre reinforced composites are developed by using traditional hot-walled chemical vapor deposition, which coats CNFs over woven CF materials, with composite lengths restricted to the magnitude of the stable hot zone [68]. The volume of the material to be coated can be maximized using rolled or scrolled woven fabric upon employing tubular furnaces [80]. Regarding the polymer composite matrix, they can be divided into two overarching classes: thermosetting and thermoplastics polymers [81]. Curing involves the development of a cross-linked molecular, three–dimensional structure in both cases. A few obligations are required to be performed as a matrix. It includes the transfer of loads between fibres, protecting the notch sensitive fibres from abrasion and framing a protective boundary between the filaments and environment [82]. Preventing assaults from moisture, chemicals and oxidation is also an essential requirement for matrix as a protective barrier [83]. The composite is needed to be provided with shear, transverse tensile, and compressive properties by the matrix. Their performance governs the behaviour of the composite under the influences of temperature [84,85].

There are a significant number of published review articles on carbon-based polymer composites that demonstrate either the use of carbon fibre, or nanocarbon particles and nanofibres as the reinforcing fillers. However, there was no emphasize on reviewing the technology that shows how the nanocarbon are coated onto CF to reinforce polymer matrices. With innovative interface-engineered hierarchical structures, this method allows CF reinforced composites to have better strength and durability. In particular, the use of micro- or nanocarbon coating on CF show the potential of using these hybrid fibres in fabricating high-performance composites for automotive [86,87] and aerospace components [88,89], and several other structural applications including construction [32] and defence [73,90]. The potentiality of such hybrid fibres for reinforcing polymer matrices are well-demonstrated by many researchers in the form of research articles, books, or patents. This article reviews those published researches and focuses on the general concepts of carbon materials coated CF reinforced polymer composite. It first summarizes the different carbon coating types onto CF. It then highlights various coating techniques such as chemical, thermal, and electrochemical. It further focuses on the different polymer matrix materials, which are essential to fabricate the polymer composites and enhance the performance of composite material. The influence of carbonaceous materials coating on CF on the mechanical properties of their reinforced composites is also discussed. Some future recommendations are also drawn based on the current limitations relevant to these hybrid fibres reinforced composite systems.

## 2. Types of Coating

### 2.1. Carbon Nanotubes and Nanofibres

**Figure 1:**
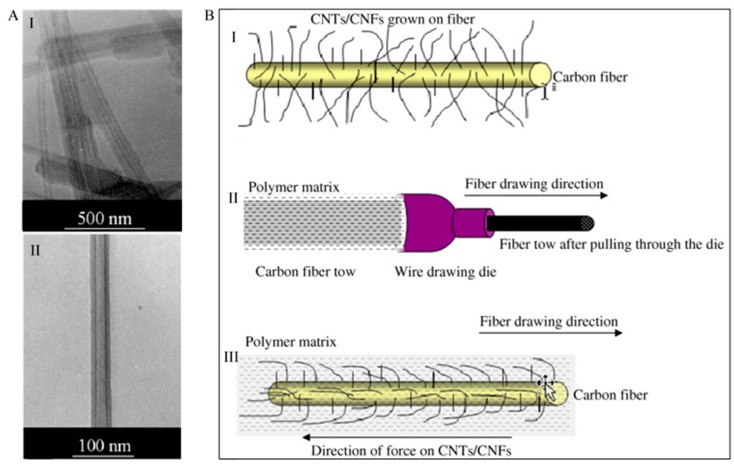
(**A**) TEM images of the carbon nanotubes: I. aligned bundles of hollow-core MWCNTs; II. isolated MWCNTs. (**B**) Schematic diagram illustrating: I. carbon fibre after the growth of CNTs/CNFs; II. fibre dipped in polymer matrix and drawn through the die to fabricate a single fibre tow composite specimen; III. carbon fibre after drawing through the die showing partial alignment of CNTs/CNFs along the fibre axis due to the viscous force of the matrix and the force due to the smallness of the orifice. Reprinted with permission from ref. [91], Copyright 2011 Elsevier Ltd.

Carbon nanotube (CNT) and carbon nanofibre (CNF) coating on CF reinforced polymer composites exhibit excellent properties that are essential in many high-performance applications including automotive [86,87] and aerospace [88,89]. High strength (>150 GPa), modulus (~1 TPa), thermal conductivity, electrical capacity and thermal stability are the common characteristics of this coating [91]. Several research works have been done based on CNT coated CF reinforced polymer composites [65,67,89]. Importantly, an optimal loading for CNT coating in the polymer composites is a key criterion to employ its outstanding mechanical characteristics in the composites. It has been studied that increasing CNT loading can primarily lead to an increase in tensile strength and tensile modulus, however, increasing CNT loading has an adverse effect on the tensile modulus and strength of the composite beyond a critical weight fraction [92]. For example, the deterioration of tensile strength, failure strain and elastic modulus of polystyrene composites has been found beyond a critical CNT mass fraction [93]. Nanotubes can cause agglomeration that decreases the interphase region and produces stress concentration sites of the composites, thereby leading to failure [94]. This can cause a reduction in the load carrying capacity of the composite as well. Therefore, it is necessary to grow CNT directly on the CFs surface. The processing parameters such as the growth time, catalyst, and growth temperature play an important role in the extent of CNT coating and the fibre surface coverage. Agnihotri et al. [95] have studied the effects of CNT coating on the CNT-coated CF/polyester composites’ properties. The researchers have found that the optimization of the multiscale composite properties can be achievable by changing the reactor duration for chemical vapor deposition (CVD). Dispersion, degradation of the CNTs and matrix viscosity are some challenging issues for CNT coating [96]. In some cases, the dispersion techniques of CNTs coating in the polymer composites resulted in low CNT graphitization, poor nanotube alignment, CNT agglomeration, inadequacy to small weight percentage (wt%) accumulations, deficiency of morphology control, and reduced matrix infusion capability while using infusion techniques, such as resin transfer molding (RTM) and vacuum assisted resin transfer molding (VARTM). The role of CF as a substrate for CNT coating permits well dispersed and high-density CNTs for the incorporation in the composite. The link among the adjoining CFs, generating electrical and thermal percolation routes throughout the composite and improving the mechanical characteristics of CF-based composites can be possible by CNTs. The interfacial adhesion between the resin-dominated (i.e., volume or weight fraction of resin is much higher than the filler) processes and the CFs can also be improved by the CNTs. The effect of sonication time on the mechanical properties of CNTs reinforced multiscale composites. Compared with neat epoxy resin, the residual heat, rate of reaction and heat flow and conversion at the beginning stage of the cure course has been enhanced by involving non-woven CNFs in the composites. Sharma and Lakkad [91] investigated the CF before and after the growth of CNTs/CNFs. According to the TEM images (Figure 1A), the centre of the CNT has a hollow core, which is very narrow. It is clear that the CNT covered the fibre surface uniformly and grew long enough with various diameter and length. As shown schematically in (Figure 1B), the long CNTs may orient themselves with the direction of drawing of the fibre when it is being pulled out through the very small orifice of the wire drawing die. The CNTs/ CNFs grown on CF is shown in Figure 1B(I) whereas the schematic diagram of polymer matrix dipped fibre and its pulling through the wire drawing die is portrayed in Figure 1B(II). The CNTs/carbon nanofibre coated CF after pulling out through the die is shown in Figure 1B(III), which displays an expected partial alignment of long CNTs/CNFs along the fibre axis, whereas the short CNTs/CNFs are still expected to remain unchanged. The cause of the alignment is due to the viscous force of the polymer matrix and also the frictional force due to the compactness of the opening of the die. Throughout the fibre pulling across the die, the exertion of the forces took place. The increase in the composites’ tensile strength is due to this partial alignment.

It has been reported that the tensile strength increased by 69% for the multiscale composites compared to the reference composites made of CF while undergoing similar heat treatment processes as the CNTs coated CF. The authors have attributed that the presence of CNT on the CF surface improved the tensile properties of the composites. It is without a doubt that the addition of CNTs influences the properties of the composites. The most simplistic route is to add the CNTs to the polymeric matrix directly before fabricating the fibre-based polymer composite. Boroujeni et al. [97] have shown noteworthy enhancements for vibration attenuation (25.8%), impact energy absorption (21.3%) and axial strain to failure (12%) by adding multi-walled CNTs of 2.0 wt% to an epoxy matrix of a fibre reinforced polymer.

### 2.2. Graphene

**Figure 2:**
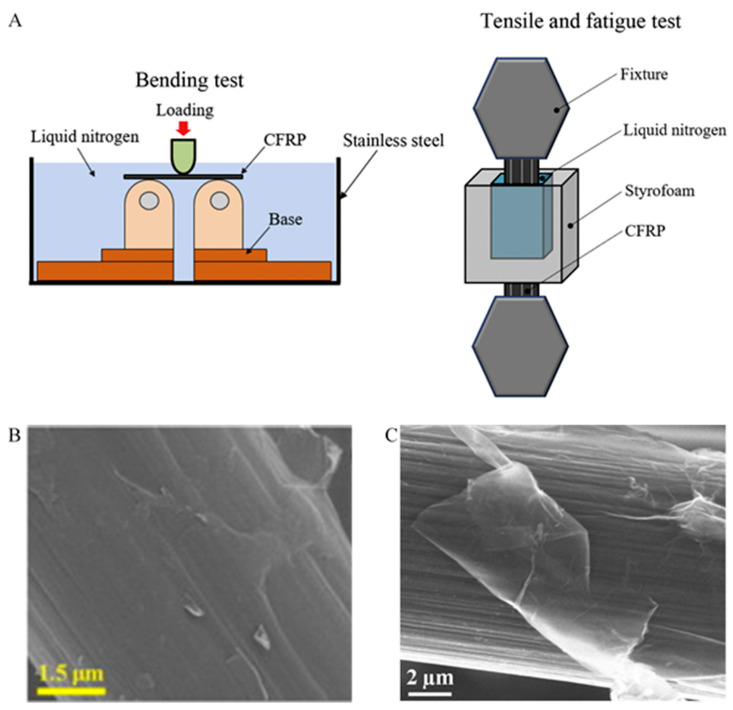
(**A**) Schematic diagrams of cryogenic testing system for three-point bending and tensile tests. Reprinted from ref. [106], Copyright 1969 Elsevier Ltd. (**B**) SEM image of GO coated SCFs. Reprinted with permission from ref. [105], Copyright 2016 Elsevier Ltd. (**C**) SEM images of graphene oxide grafted on CF. Reprinted with permission from ref. [107], Copyright 2020 Elsevier Ltd.

Graphene coated CF reinforced polymer composites are used due to graphene’s exceptional electrical, thermal, and mechanical properties [98,99,100]. Numerous studies have been done by incorporating graphene or graphene oxide (GO) sheets onto a wide range of polymer matrices [98,101,102,103]. He et al. [104] have introduced the graphene/CF hybrid as a multi-functional interfacial nano-reinforcement of CF reinforced polytetrafluroethylene composites for the enhancement of the mechanical and electrical properties. A slight increase in the tensile strength was also observed. Accordingly, an effective method is necessary for depositing graphene oxide onto the surface of CFs. For optimum improvement in mechanical properties of polymer matrix composites, the major key issues for favorable interaction are the alignment and dispersion of graphene on the polymer and the surface modification. Agglomeration of the graphene sheet is often caused by the entangled structure produced during the synthesis of graphene and interlaminar Van der Waal forces. While mixing with polymer, major complications arise due to the dispersion and peeling of graphene in media. Poor agglomeration and dispersion of graphene sheets can trigger nano defects in laminated composites, which can lead to poor enhancement of the mechanical properties. Multifunctional sizing agents such as graphene oxide can be consistently dispersed and robustly adsorbed on the CF surface to form an additional hierarchical reinforcement. It has the capability to generate in enormous numbers at a relatively low cost and holds outstanding mechanical properties. For the time being, it covers numerous oxygen functional groups, for example; epoxide, hydroxyl, and carbonyl groups. It has been studied that graphene oxide treatment on the surfaces of short CF can significantly enhance the adhesion at the interface of CF/polyethersulfone composites owing to the hydrophilic oxygen-functional groups in the basal planes of graphene oxide. Li et al. [105] has examined graphene coated short CF reinforced polyethersulfone composites to be used in cryogenic engineering field. The cryogenic mechanical performance was examined in the cryogenic tensile and bending test systems (Figure 2A) as demonstrated by Okayasu et al. [106]. In the work conducted by Li et al. [105], the sizing was conducted in a method similar to the Hummers method through acid oxidation of graphite powders. The preparation of pure polyethersulfone was conducted using the injection molding technique, which helps to observe the effect on the mechanical properties of the polyethersulfone matrix when an extrusion compounding process is used. Dried graphene oxide-coated short CFs were re-dispersed in deionized water and intensively stirred. The coating of short CFs was conducted in order to assess the coating efficiency of graphene oxide on short CFs in the physical adsorption process. According to Figure 2B, the graphene oxide was still robustly adsorbed on the short CFs surfaces, as shown in the SEM image of the collected short CFs. The researchers have reported an increase in tensile and flexural moduli when graphene oxide coating is used due to their outstanding elastic modulus. The optimal graphene oxide content of 0.5 wt% can efficiently improve the overall mechanical performance of the composite. Moreover, the usage of reduced graphene oxide can improve the interfacial property and mechanical properties of CF. The reduced graphene oxide coated CF has been found to be more effective than CF to enhance the unsaturated polyester-based composites in terms of electromagnetic interference shielding property. Chen et al. [98] have obtained that the shielding effectiveness of the composite at the frequency range 8.2–12.4 GHz (x-band) of reduced graphene oxide-based CF having 0.75% mass fraction can reach 37.8 dB, which is a 16.3% increase compared to that of CF unsaturated polyester based composite (32.5 dB) for the same mass fraction. Directly grafting graphene on CF can contribute to enhancing the mechanical properties of polymer composites. Figure 2C shows the SEM image of grafted GO on CF by ester linkage in a low temperature (>100 °C) solvothermal process [107]. Zhang et al. [108] have studied the novel hierarchical reinforcement of CF on which graphene oxide is directly grafted. The interphase strength between the CF and resin matrix was boosted due to the grafting of graphene oxide onto CF. Surface modification can effectively increase the polarity and wettability of the surface of CF without compromising the tensile strength [1]. The efficient graphene oxide reinforcement between CF and matrix resin results in these improvements. The ample potential in high-performance CF polymer composites is demonstrated due to the enhanced mechanical performance of this kind of hierarchical reinforcement.

### 2.3. Carbon Black

**Figure 3:**
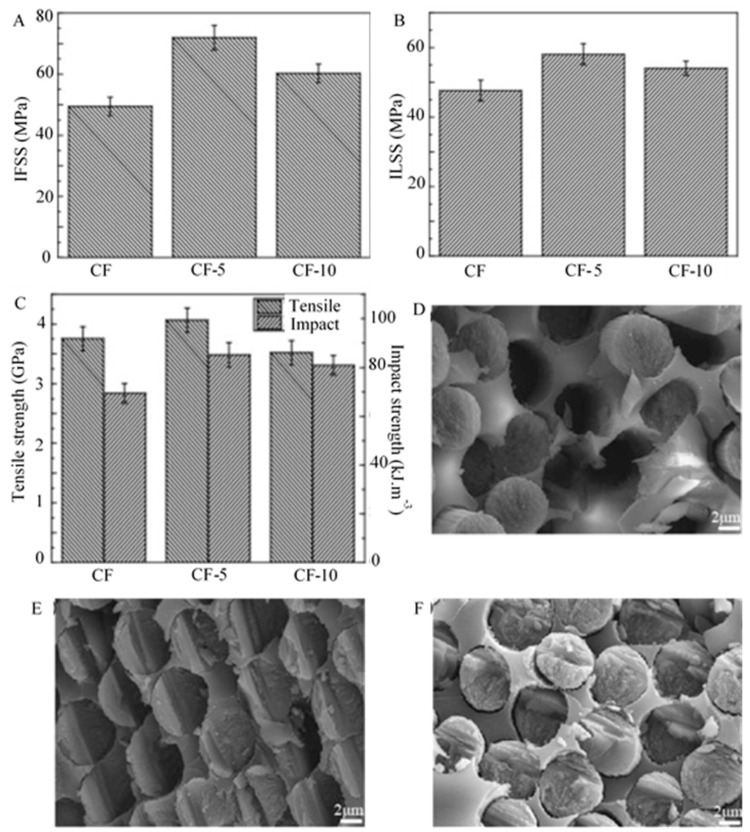
Mechanical properties of carbon black coated CFs/epoxy composites: (**A**) IFSS tests results of the composites. (**B**) ILSS of the composites. (**C**) Impact test results of the composites and single fibre tensile strength. SEM morphologies of the fracture surface of composites (**D**) the untreated-CF, (**E**) CF-5 min, (**F**) CF-10 min. Reprinted with permission from ref. [22], Copyright 2017 Elsevier Ltd.

The usage of carbon black coated CF reinforced polymer composites has been considered in some researches due to their microcrystalline structure and different functional groups available on the carbon black surface [22]. Comprising virtually pure elemental carbon, carbon black is found in the form of colloidal particles (pellet or powder) synthesized by incomplete combustion or thermal decomposition of gaseous or liquid hydrocarbons [76]. The carbon atoms have hexagonal planes, resembling that of graphite, and the arrangement of these atoms of carbon black crystallite is ordered in graphite layers individually [109]. However, quasi-graphite crystals have a disordered array of carbon atoms in the adjoining layers. A high temperature is required for the manufacturing process of carbon black [110,111]. The primary structure is formed by the generation of adjoining particles simultaneously, melted into a chain, and occupying three degrees of space. This primary structure forms loose secondary structure steadily because of the Van der Waals force or physical adsorption. These secondary structures can also be called temporary structures due to their susceptibility to mechanical damage during processing. Dong et al. [22] applied a carbon black coating on the surface of CF to enhance the mechanical properties of CF/epoxy composites. The interfacial strength between resin matrix and CFs was calculated using interfacial shear strength (IFSS) tests in this study. As shown in Figure 3A, IFSS of 5 min modified CF (CF-5) increased by 44.4% (49.45 MPa to 71.91 MPa) compared to untreated CF (CF). The surface energy was enhanced by the homogeneous distribution of carbon black, leading to enhanced wettability between CF and epoxy resin. The development in the surface roughness leads to an increase in bonding between the fibres and resin. Interface shear strength of 10 min modified CF (CF-10) significantly rose to 54.05 MPa at increasing carbon black growth time. The variability of the secondary structures of carbon black created caused the microdroplet composites to slide with relative ease from the fibre surface, even though the roughness and wettability of the fibres were improved. The properties of polymeric matrix and CFs as well as the efficacy of the interfacial adhesion between the matrix and the CFs are completely responsible for the composites’ outstanding mechanical properties. Figure 3B illustrates the correlation between the different growth time of the carbon black and its effect on the CF/epoxy composites’ mechanical properties. The interlaminar shear strength (ILSS) of untreated CF composites was observed to be 47.6 MPa, however, the shear strength value of CF-5 min was 22.0% and CF-10 min was 13.55% higher compared to that of untreated CF composites due to the weak bonding between fibre and matrix. The structural integrity of composites was improved by the carbon black deposited on the CF surface that shifts the load successfully from the matrix resin to the fibre. The defects on the fibre surface coverage and stress concentration reduction can be possible by the carbon black coating, thereby increasing composites’ mechanical properties (Figure 3C). The carbon black’s secondary structure deteriorated the interfacial adhesive force between the resin and the fibres with time. The deposition of carbon black on the untreated-CF, CF-5, and CF-10 can be seen in Figure 3D–F. There was an enhancement in the surface energy of CF and in the wettability between the carbon black and matrix. As no holes were reported in the composite and epoxy matrix, we can say that the mechanical properties and interfacial adhesion of CF reinforced matrix were considerably enhanced. Several gaps between the fibre and the resin were detected while decreasing the number of voids and drawn fibres. Collectively, carbon black can increase the surface energy, the wettability, the CF surface roughness and cover the defects of CF surface [22].

### 2.4. Graphite

**Figure 4:**
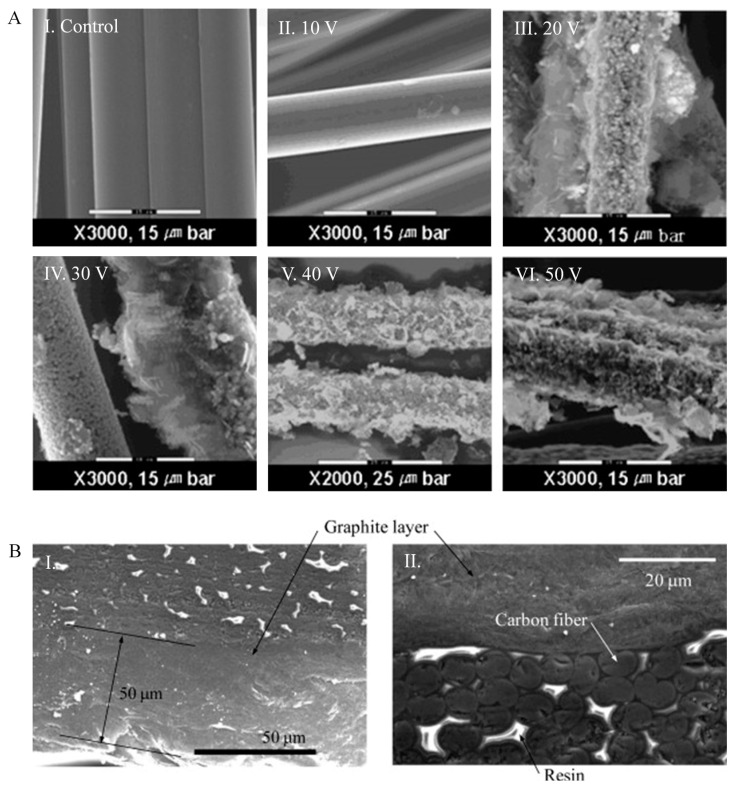
(**A**) SEM images for the surface of CFs at different applied voltages for 5 min. Reprinted with permission from ref. [112], Copyright 2008 Elsevier Ltd. (**B**) SEM images: I. composite plate coated with a 2-μm-thick graphite layer; II. composite plate coated with a 50-μm-thick graphite layer. Reprinted with permission from ref. [116], Copyright 2011 Elsevier B.V.

Graphite coated CF reinforced polymer composites graphite are gaining interest because graphite is considered in nature as the stiffest material and has outstanding electrical and thermal conductivity owing to its layered structure. Exfoliated graphite nanoplatelets (xGnP) can be formed from graphite flakes interpolated with extremely concentrated acids which, at high temperatures, can be increased over a hundred times over their initial volume. The particles can be made of singular or a few layers of graphene sheets with considerable exfoliation. Park et al. [112] have fabricated xGnP/Cu coated CFs epoxy composites. Excellent electrical and thermal conductivity and good mechanical properties of xGnP polymer nanocomposites were found at low xGnP content of even less than 2 vol%. Due to the high surface area and aspect ratio of xGnPs, a percolated conducting network within the polymer is generated with a concentration of less than 2 vol%, thereby resulting in outstanding properties of these polymer nanocomposites. In Figure 4A, the SEM images of the coated CFs with varying applied voltages using the copper plates as anode are shown. As shown in Figure 4A(II), except at the applied voltage of 10 V for 5 min, the surface of CFs treated with different process conditions was seen to have a relatively good deposition of xGnP with a size of 1 μm on the CFs in the ESEM images. As shown in Figure 4A(V,VI), there is redundant deposition above 40 V for 5 min. It is provident that the quantity of xGnPs on CFs steadily rises with an increase of the voltage applied at constant deposition time. Further investigation of the morphology of the coated fibres specified that there were additional constituents such as granular and co-deposited particles, as signified in Figure 4A(III,IV), other than xGnP. It has also been found that the xGnP/Cu coated CF reinforced epoxy composites’ flexural strength and modulus are greater because of the coating of xGnP/Cu on the CFs, which has a reinforcing effect. In one study, xGnP coated CFs by using a solution of xGnP was produced, and a 19% increase in interlaminar shear strength was obtained with 3 wt% xGnPs [113]. In another research, the thermal conductivity was 13% higher for xGnP coated fibre with the addition of 1 wt% of xGnP [114]. Kostagiannakopoulou et al. [115] showed that there is a 176% increase in thermal conductivity in nanomodified polymers and a 48% increase in thermal conductivity for CF reinforced polymer composite with the addition of 15 wt% GnP into the epoxy matrix. Due to its conformability, graphite has a very low contact resistance, which is why, for composite bipolar plates, methods of graphite coating were developed. Yu et al. [116] have conducted a study for polymer electrolyte membrane fuel cell based on graphite-coated CF epoxy composite bipolar plate. In this research, the cross-sections of the composite plate surface were shown (Figure 4B) when the thicknesses of the graphite layer are 2 µm and 50 µm (Figure 4B). It was found that the graphite layers with a thickness of 2 µm and 50 µm coating on the carbon/epoxy composite bipolar plates had 14% and 10%, respectively, of the total electrical resistance compared to that of the composite bipolar plate without surface treatment under 1 MPa of compaction pressure. This proves that the composite bipolar plate coated with graphite would be an appropriate substitute to metal bipolar plates.

## 3. Coating Techniques

### 3.1. Chemical Vapor Deposition

**Figure 5:**
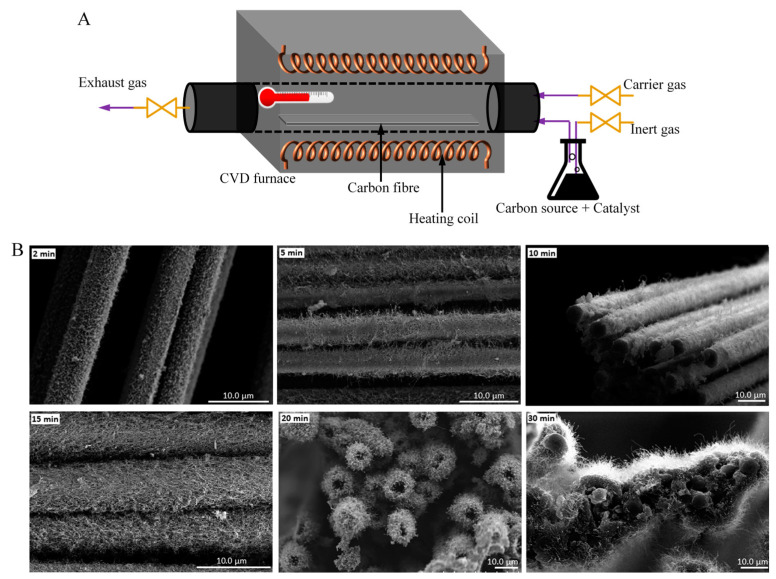
(**A**) Schematic diagram of a floating catalyst CVD. (**B**) SEM images of CNT morphology on CF surface as a function of time under 700 °C temperature and 100 mL/min carrier gas flow rate. Reprinted with permission from ref. [117], Copyright 2014 Society of Plastics Engineers.

Chemical vapor deposition (CVD) is considered to be the most effective method used to grow carbon nanofibres (CNFs), CNTs (CNTs), and graphene [65,66,67,68,69,70,71]. The critical parameters of this deposition technique such as growth duration, growth temperature, catalyst concentration, and flow rate of carbon source gas can be changed to obtain CNFs and CNTs of varying structures and morphologies. For instance, Ghaemi et al. [68] investigated the effects of the amount of CNF coating on CF and thickness for the improvement of polymer nanocomposites on the mechanical properties. For this purpose, the synthesis of CNFs on the surface of the CF was conducted using the CVD method under varying process conditions, and afterwards, these nanofibres have been used to fabricate the composite and as fillers in a polypropylene matrix. During the experiments at various temperatures, three of them were considered, including keeping the catalyst concentration at 100 mM, reaction time for 30 min, and flow rate of acetylene at 50 sccm fixed and varying reaction temperatures of 450, 550, and 650 °C. A dramatic effect on the CNF growth was seen due to the reaction temperature of the thermal CVD method. It can be understood that the graphitization and morphology of the carbon nanoparticles have been influenced by the temperature. However, this technique requires high temperatures (>500 °C) and flammable gases (carbon feedstock, hydrogen), so this method is limited to small-scale batch processing. In many research works, developing a one-step coating process is necessary, especially in the case of CNTs, as they grow straight onto the CF without the requisite for the pre-treatment for CF or purification of CNTs after treatment. Different chemical vapor deposition techniques such as catalytic or thermal, plasma enhanced, and floating catalyst methods have been introduced to fabricate single-walled CNT and multi-walled CNT. In the existence of catalysts, organic compounds are decomposed. The development of CNTs occurs onto the substrate in the catalytic and plasma enhanced method. A significant amount of CNTs can be produced without the use of any substrate. Hence, the floating catalyst method is highly economical for synthesizing CNT on a large scale (Figure 5A). This method has been approached by many researchers. Aziz et al. [67] conducted a study where the synthesis of CNTs was conducted using a floating catalyst CVD method on the high-performance CFs surface. This method provides a means to modify a composite’s mechanical properties on the fibre-resin interface. In addition to dictating CNT morphology with processing temperature, Aziz et al. [117] also found that the growth time is another important variable that can be used to both understand the growth process and tailor the interfacial properties of the resulting composite assembly. Figure 5B displays a set of images showing CNT morphology as a function of growth time for 2, 5, 10, 15, 20, and 30 min at 700 °C reaction temperature and 100 mL/min hydrogen flow rate. The highest extent of CNT coating on CF has been found at a chemical vapor deposition at 700 °C temperature and a reaction time of 30 min. As comparatively new research, Karakassides et al. [71] fabricated radially aligned graphene nanoflakes, grown directly on carbon fibres via a simple one-step microwave plasma assisted chemical vapor deposition method. The hybrid fibre gave tremendous increase in mechanical and electrical properties when reinforced in epoxy matrix.

Nevertheless, monitoring the size and configuration of the catalyst nanoparticles is a key problem for this method. It is desirable to confirm their aptness to process CNT, CNF, or graphene coated CF polymer composites. In this regard, CNTs have been developed directly, using thermal chemical vapor deposition, on the surface of CFs as well as fabric as described by Bedi et al. [118,119]. Using this technique, a stronger interface was found between the CNT coated CF and epoxy. This technique is suitable for the aligned and controlled structure of CNTs. In another research, the elementary concepts of CNT development by catalytic CVD technique were discussed [120]. The nucleation of the CNTs took place in the clusters of catalyst and CNT filament development started at the desired temperature. The growth of filament remains due to the incessant stock of ferrocene–toluene mixture inside the reactor. By increasing the reaction time from 1 to 2 h the filaments started growing in a curled or twisted form. As stated, enhanced structural properties can be possible in CNTs-polymer composites upon applying this technique.

### 3.2. Spray Coating

**Figure 6:**
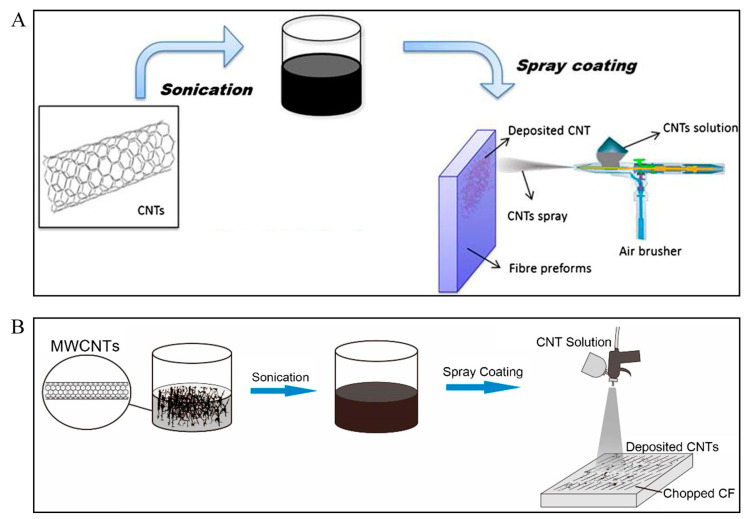
Schematic illustrations of CNT spray coating. (**A**) Onto CF prepreg. Reprinted with permission from ref. [121], Copyright 2014 Elsevier Ltd. (**B**) Onto chopped CFs. Reprinted with permission from ref. [32], Copyright 2019 Elsevier Ltd.

Spraying methods are considered as the simplest procedures to deposit and scatter nanomaterials onto substrates. The utilization of strong airflow to produce very tiny droplets can produce a spray that can be distributed onto films, fibres, or fabrics directly. Zhang et al. [121] achieved enhanced mechanical properties by utilizing this technique for CNT deposition on CF prepregs (Figure 6A). This technique provides a means to introduce CNTs into fibre composite laminates and has a key benefit over conventional methods on the basis of CNT modified resins. Spraying the CNTs can be restricted to zones susceptible to damage rather than dispersed throughout the entire composite structure. Consequently, this technique permits the formation of truly hierarchical nanoengineered composite systems with custom-made CNTs localization for the enhanced composite performances. Additionally, it develops the viability of scale-up in industrial applications for the greatly successful usage of nanofillers. Among the spray coating processes, thermal (flame) spray techniques are used extensively to deposit thick coatings so as to enhance the surface of components made from various types of materials. This technique comprises of a group of coating methods wherein a source of heat is involved to melt the material in the form of powder, wire or rod and propel the molten or semi-molten particles by intensifying the process gases toward a prearranged substrate to generate a build-up of coating. The spray techniques were limited to be applied only on metal alloys, cermets and several low-performance polymers to produce a defensive coating on the substrate. It forms a high-performance coating on parts or regions that would be difficult or expensive to be coated by other methods. This is considered as one of the main benefits of this spraying coatings. It is recommended to use this technique to deposit a metallic coating on the surface of fibre reinforced polymer even if there are some challenges. During the spraying process, the protection of materials of fibre reinforced polymer against erosion, fracture of fibre and thermal degradation can be possible by adding filler particles layer as a layer on the top surface or as a bond coat. The improvement in the adhesion of the coating/fibre reinforced polymer and the decrease of the thermal and mechanical degradation of the substrate throughout the spray process are the major objectives for the extension of spraying processes as a manufacturing method for the coating of fibre reinforced polymer composite. Hu et al. [32] have demonstrated the CNT deposition onto the CF surface (Figure 6B) and then compounded into CF reinforced high-density polyethylene (HDPE) composites by the process of extrusion and injection molding. The surface morphologies of raw CFs were level or smooth. The morphology of CFs had been significantly changed with surface modification, with the deposition by spray coating of CNTs onto the surface of the fibre. Li et al. [122] observed that the surface modification of CFs yielded similar results. In this research, commercial carbonxylic- and hydroxyl-functionalized CNTs were chosen to coat the CFs of similar lengths by utilizing an aqueous suspension deposition method. Overall, the spray coating of CNTs onto CF exhibited a promising method of surface modification, which can be used to fabricate the composites with enhanced interfacial properties to improve the mechanical performance of the hierarchical composites.

The spray coating technique can also be useful to coat graphene materials on CF for composite reinforcements [123,124,125]. Altin et al. [123] modified the surface of CF with graphene oxide through the spray coating method. The CF reinforced epoxy matrix composites were prepared by hand layup technique, and it was observed that graphene oxide particles were homogeneously coated on the surface of the carbon fibres. Young’s modulus and tensile strength of the composite were found to be significantly increased when CF was coated with graphene.

### 3.3. Electrophoretic Deposition

The electrophoretic deposition technique has received widespread attention due to its capability to fill great amounts of nanomaterials on complex, three-dimensional surfaces with a short process time to confirm high levels of flexibility. Charged nano reinforcements can be promptly coated on electrically conducting CFs through this technique under an electric potential. CNFs, CNTs, aramid nanofibres, and graphene oxide sheets have been effectively added onto CFs for making reinforced polymer with improved interfacial properties using this method [73,74,75]. In one study, the researchers coated CNTs onto the surface of CFs using this technique and produced multiscale hybrid composites that showed an enhancement of ~17% of the interlaminar shear strength [74]. Pre-treatment is required to incorporate electrically charged functional groups while using CNTs. The weakening of CNTs is unavoidable since covalent functionalization of CNTs requires carbon sites that are non-conjugated to create covalent bonds with the preferred functional species. Unfortunately, major works related to CNT coated fibre composites have illustrated the chemically modified CNTs to coat on the fibre surface. This is caused by the well-dispersed CNTs before coating, which develops electrical charges in the solution. In fact, covalent functionalization is responsible for the unavoidable inherent deterioration and degradation of CNTs. Kwon et al. [88] presented the preparation of graphene oxide/CNTs hybrid materials and coating on CF surfaces using this technique to enrich both the mechanical and electrical properties of the CF reinforced polymer. Both the quantity and thickness of this hybrid fibre on the CF surface were tailored by controlling electrochemical deposition conditions, for example, the applied voltage, time, the solution concentration, and the electrodes distance. The quantity of this hybrid fibre onto the surface of the CF became negligible while proceeding with the deposition for 1 min at low voltage (5 V). By increasing the time of application to 2 min and the applied voltage to 10 V, correspondingly, these hybrids were coated on individual CFs homogeneously. This deposition technique can be described as a simplistic and easy process where the coating amount and the surface morphology can be regulated and is very efficient in terms of coating the CF surface with graphene/CNT hybrids. Sui et al. [126] have demonstrated the CNT-toughened transition layer on the carbon hierarchical fibre/epoxy composites via electrophoresis deposition method. Oxidized multiwall CNTs were built to enhance the interfacial and fatigue-resistant performance of CF/epoxy composites synergistically. As found, the hierarchical composites exhibited an increase of 33.3% in interfacial shear strength, 9.5% in flexural strength, 10.5% in interlaminar shear strength, and after fatigue tests, a 4.5% improvement in residual bending strength retention. Overall, this technique is a successful and multipurpose method for the deposition of CNTs onto the CF surface that can be speedily automated and applied for industrial applications.

### 3.4. Dip Coating

Dip coating is considered one of the most cost-effective and facile approaches for the deposition of nanomaterials on a target substrate [70,72]. It describes the removal of a substrate from a liquid coating medium that deposits a wet liquid film. A consistent liquid film is entrained on the removal of the substrate from the coating of the fluid that combines by accompanying chemical reactions and drying. Additional curing is required to achieve the final material of coating. The basis of the film formation is a fluid mechanical equilibrium between the entrained film and the receding coating liquid. The equilibrium is regulated by some forces. Of them, the viscous drag and the gravitational force are the most important. However, different forces such as surface tension, inertial force or the disjoining press play a critical role as well. This technique can also be useful for complex shapes to be coated with modified techniques. Although this is a classic batch process, it can also be operated as a semi-uninterrupted process. The material of the substrate usually has to be considered by the chemical resistance to the solution of coating and the heat resistance to endure the consequent curing or post-treatment. Awan et al. [127] prepared the CNT coated CF reinforced epoxy matrix multiscale composites. By using dip coating and spray-up processes, the deposition of multiwalled CNTs on micrometer-sized CFs was obtained. Non-uniform coating of CNTs along with the presence of their clusters and other carbonaceous impurities were also noticeable through the spray-up process. An even coating of CNTs was attained after the dip coating process. Since dip coating exhibited a better deposition quality, 12 sheets of CFs were coated with multiwalled CNTs and subsequently filled with epoxy resin to make multiscale composites. The researchers observed a 14% increase in interlaminar shear strength in composites containing CNTs as compared to reference composites. In several research works, CFs were dip-coated with graphene oxide and used to fabricate polymer composites [70]. Since the implementation of CF reinforced composites is reliant to a great degree on the properties of the fibre/matrix interface, it is important to improve the interfacial properties in CF-based epoxy composites. The sheets of graphene oxide are arbitrarily distributed, adjoining the individual fibre surfaces by treating them with the modified sizing agent of graphene oxide. Kwon et al. [88] found an enhancement of the interfacial shear strength of those composites of 70.9% compared with that of the virgin CF composites and 36.3% compared to commercial, sizing agent modified CF composites. Importantly, there was an improvement of 12.7% of the interlaminar shear strength when graphene oxide sheets were incorporated into the interfacial regions of matrix and CFs compared to commercial-sizing-modified CF composites. Tensile strength and tensile modulus were also obtained to be greater than that of the normal composites. The researchers attributed that this technique is an effective means to improve the interfacial and tensile properties of the composites.

## 4. Matrix Materials

### 4.1. Thermoplastics

**Figure 7:**
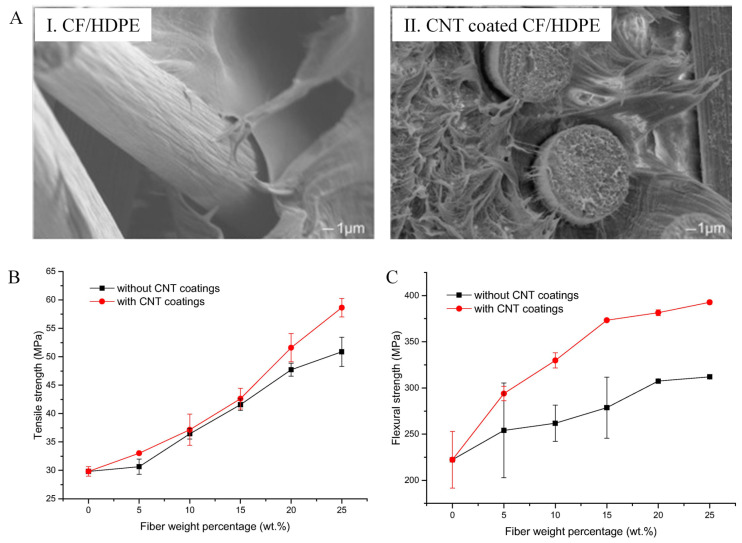
Effect of CNT coating on morphological and mechanical properties of CF reinforced HDPE composites: (**A**) Fracture morphologies: I. Neat CF/HDPE composite; II. CNT coated CF/HDPE composite. (**B**) Comparison of tensile strength. (**C**) Comparison of flexural properties. Reprinted with permission from ref. [32], Copyright 2019 Elsevier Ltd.

Thermoplastic matrices along with CFs have increasing significance in their application since they have a high distortion temperature [128,129,130,131,132,133,134]. The composite strength with more or less perfect continuity is often controlled by the fibre/matrix interface. The polymer normally acts as the matrix, while CF acts as the discontinuous phase in CF reinforced composites. Thermoplastics can be classified into common plastics, for example, polyethylene (PE), polypropylene (PP), acrylonitrile butadiene styrene (ABS) resins, engineering plastics, such as polycarbonate (PC), polyamide (PA), polyetheretherketone (PEEK), polyetherimide (PEI), polyphenylene sulfide (PPS), and polyethersulfone (PES). The lack of a curing stage and their less hazardous chemical compositions, enhanced recycling suitability and mass production proficiency in comparison with conventional thermosetting resins can be found in thermoplastics. Therefore, it has been gaining attention as polymer matrices. Conventional molding procedures, such as rotational molding, injection molding, extrusion molding, vacuum forming, and compression molding are usually used to fabricate thermoplastic resins. In several studies, the researchers have investigated PP as a thermoplastic matrix to develop CNT coated short CF reinforced polypropylene hybrid composites [32,67,117,135,136,137]. PP is a member of conventional thermoplastics and is produced in large amounts. It is not very susceptible to chemical stress cracking. For these reasons, CF reinforced PP composites have widespread applications that include body constructions and interior trim for vehicles, fuel cells, fuel tanks and several other mechanical uses. Hu et al. [32] demonstrated the development of hierarchical CNT-based CF reinforced high density polyethylene (HDPE) composites. HDPE is considered a potential thermoplastic material for its great impact strength, low friction coefficient, low density, outstanding chemical resistance, and suitable fatigue resistance. Researchers have investigated the influence of CNT coating on the extrusion of CF reinforced thermoplastic composites. To deposit CNT on the surface of CF, spray coating technique was used while extrusion and injection molding employed for CNT coated CF reinforced HDPE was used. As shown in Figure 7A(I), poor interfacial adhesion was found in the uncoated CF reinforced HDPE composites. In contrast, the difference between CFs and HDPE matrix became considerably reduced for the CNT coated HDPE composites than those counterparts that were uncoated, as shown in Figure 7A(II). The noticeable growth of toughness inside the HDPE matrix can be recognized when introduced with a very low amount of CNTs. Several CNTs were removed from the fibre surface and dispersed into the matrix to generate the toughness of the polymer matrix throughout the twin-screw extrusion of hierarchical CNT coated CF HDPE composites. Consequently, it encouraged the release of stress concentrations and hindered the propagation of the crack to form a stronger interphase. The better fibre/matrix adhesion has increased the tensile strength (Figure 7B) and flexural strength (Figure 7C) of the composites when the reinforcing CFs were coated with CNTs [32].

Another special engineering thermoplastic is polyethersulfone (PES); an amorphous, amber-colored, pristine material. It exhibits relatively large mechanical strength, has a high glass transition temperature (T_g_, ~225–230 °C) [138,139], great dimensional and outstanding thermal stability, and chemical resistance. Moreover, it is less susceptible to creep, has great flame retardancy, outstanding insulation property and great dielectric strength. In addition, CFs, glass fibres and graphene oxide are added to PES to create composites with improved mechanical properties. CF reinforced PES composites have been extensively employed in fields such as the automobile and aerospace industries, sports, off-shore- technology, and chemical engineering areas because of their exceptional mechanical and physical properties. Li et al. [140] introduced the graphene oxide coated short CF reinforced PES composites by using the melt blending and injection molding methods. The researchers found that the graphene oxide coating on short CFs can enhance the short CF and PES interfacial adhesion. As a result, the mechanical performance of the composite can be augmented by improving short CF reinforced PES composites.

### 4.2. Thermosets

CF reinforced thermoset composites have been broadly incorporated in numerous advanced technological fields that include wind power, aerospace, and transportation. in current decades [141,142,143,144,145,146,147,148,149]. These thermosetting resin matrix composites are categorized by their specific strength and stiffness, stability of the geometry, and designability. They have a permanent three-dimensional, cross-linked network structure, good mechanical strength, thermal stability, and chemical resistance. After crosslinking, they cannot be melted. In general, the mixture is put into a mold to be heated and forms crosslinks by blending the resin with different additives and reinforcements. These polymers become solidified at room temperature and can be re-melted since crosslinks are formed. Epoxy resins and their composites are most commonly utilized in aerospace constructions among all thermosetting resins. They have excellent chemical and mechanical properties, low shrinkage, and adhere sufficiently to most fibres. Some factors such as physical properties, higher temperature performance, and environmental resistance need to be considered for this adhesion to the fibre. Epoxy materials can withstand high temperatures (>200 °C). This is the most widely used polymer matrix for CFs. Due to their highly aromatic behavior, the benefits of their strength and their stiffness can be used in the composite. Epoxies can be framed in a range of properties to serve as materials for the matrix for CF reinforced polymers composites. The relevant composites are versatile and can be used in numerous engineering structure applications, for example, anchors for concrete, crack injection, and bond precast concrete elements. Additionally, such composites have outstanding corrosion resistance and less shrinkage once cured. Accordingly, these composites have a low tendency to cracking in thermal loads. Similar to reinforcing thermoplastic resins, carbon materials coated CFs can also be used to make epoxy-based composites. Many reports used epoxies as the matric material and CNT coated CF and fabric as hybrid reinforcements [58,69,123,150,151,152,153,154]. Most significantly, CNT coating provides high fibre/matrix anchorage and consequently increases the mechanical, electrical, and thermal properties. Comparatively new field of research works used graphene coated CF for reinforcing epoxies. Karakassides et al. [71] examined the growth of graphene nanoflakes (GNFs) onto CFs as the reinforcing interface for epoxy composites. GNFs were grown via microwave plasma enhanced CVD method. 800 W microwave power and 600 °C reaction temperature was found most suitable for making GNFs/CF fibres with the best interfacial adhesion with the surrounding epoxy matrix. Compared to bare CF, a 28% improvement in the tensile strength was seen for the hybrid fibres through single-fiber tensile strength tests, while the interfacial shear strength was augmented by 101.5%. Using the similarly fabricated fibres, the electrical conductivity of the composite was enhanced by 60.5% for yarns and 16% for single fibre as well as a 157% improvement in the electrochemical capacitance for yarns. The researchers have found improvement of the interfacial strength between the graphene nanoflakes and epoxy resin. Altin et al. [123] investigated graphene oxide-modified CF reinforced epoxy composites. As examined, the neat CF-epoxy interface on the fractured surface of the composite is comparatively weak, while the interface of the fractured graphene oxide modified CF reinforced composites samples is reasonably better. Due to similar chemical structure, graphene oxide makes stronger chemical bonds through functional groups such as epoxy on the graphene oxide surface with the epoxy matrix. Indeed, a strong interface adhesion promotes the effective load transfer from the matrix to the fibre by decreasing the concentration of the tension, consequently intensifying the structural integrity in composites. Thus, fibre-thermoset matrix resin has the potential to guarantee more effective properties such as delamination resistance, interlaminar shear strength, fatigue, and corrosion resistance in composites due to their strong interfacial interactions.

## 5. Conclusions and Future Recommendations

**Figure 8:**
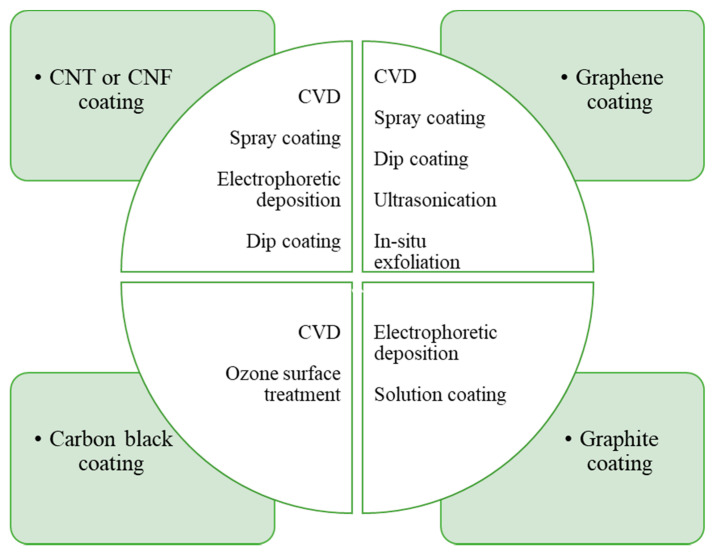
Carbon materials and techniques used for CF coating to fabricate polymer composites.

Carbon materials coated CF reinforced polymer composites are now being applied in the automotive and aerospace industries on an extensive scale worldwide due to their attractive mechanical and physical properties. New polymers, coating materials, and processing techniques are continuously being developed for these composites. Researchers are focusing on improving coating techniques, and the bonding between fibres and matrix materials. In this review, we have discussed the variation in coating material such as CNT, graphene, carbon black, and graphite for these fibre reinforced composites. Since CNTs have exceptional mechanical and physical properties, it becomes the most desirable coating material to enhance the insufficient properties of these composites among all coating materials. They can impart the required properties to the composites for accurate control on the process of their synthesis to align them in the optimal direction. They can increase tensile strength and modulus of the composites. Consideration of critical mass fraction is important to avoid the adverse effect on failure strain, tensile strength, and elastic modulus of the composites. It is also indispensable to grow CNT directly onto the surface of CFs by considering parameters such as growth time, catalyst, growth temperature, etc., to obtain the suitable quality of CNT coating. We have also described several research works based on different coating techniques for these reinforced composites. Direct coating methods that include chemical vapor deposition, spray-up methods, electrophoretic deposition, and dip coating have been discussed. Figure 8 shows the carbon coating materials and techniques used so far by the researchers. Of the coating techniques, CVD and spray-up methods are the most utilized techniques for direct coating on fibre surfaces. In this review, different matrix materials comprising thermoplastic and thermoset have been explained. This entails knowledge of the matrix material, its behavior, and its utilization in the composite field.

Although the use of carbon materials coated CFs are widely investigated by many researchers, there are a few limitations that are needed to consider regarding fibre coating and/or composite fabrication. For instance, the agglomerative nature CNTs could cause ununiform coating onto CFs, which eventually results in poor and random interfacial bridging with the matrix material. Moreover, clusters of CNTs could also be separated from the CF surfaces while a tensile or bending load is applied to the composite, providing premature failure of the composite system. In the case of graphene coating on CF, the 2D nature of graphene hinders the formation of a real 3D distribution of the reinforcing fibres. Similar to CNTs, graphene has a large surface area and tends to agglomerate due to the strong Van der Waal cohesive force. This could also result in the delamination of CF-matrix interfaces. Overall, all the coating techniques investigated so far are batch processes and incorporate high costs. The particulate forms of carbon materials are also harmful to the environment when exposed. To overcome the abovementioned limitations, further research on carbon materials coated CF fibre should be conducted and their usability for polymer composite reinforcements should be trialed in high-volume production facilities.

## Data Availability

Not applicable.

## References

[1] Mirabedini A., Ang A., Nikzad M., Fox B., Lau K.T., Hameed N. (2020). Evolving strategies for producing multiscale graphene-enhanced fiber-reinforced polymer composites for smart structural applications. Adv. Sci..

[2] Njuguna J. (2013). Structural Nanocomposites: Perspectives for Future Applications.

[3] Rajak D.K., Pagar D.D., Menezes P.L., Linul E. (2019). Fiber-Reinforced Polymer Composites: Manufacturing, Properties, and Applications. Polymers.

[4] Soutis C. (2005). Carbon fiber reinforced plastics in aircraft construction. Mater. Sci. Eng. A.

[5] Lewis S. (1994). The use of carbon fibre composites on military aircraft. Compos. Manuf..

[6] Tong Y. Application of new materials in sports equipment. Proceedings of the 2nd International Conference on Frontiers of Materials Synthesis and Processing.

[7] Friedrich K.M., Almajid A.A. (2012). Manufacturing Aspects of Advanced Polymer Composites for Automotive Applications. Appl. Compos. Mater..

[8] Liu L., Jia C., He J., Zhao F., Fan D., Xing L., Wang M., Wang F., Jiang Z., Huang Y. (2015). Interfacial characterization, control and modification of carbon fiber reinforced polymer composites. Compos. Sci. Technol..

[9] Drechsler K., Heine M., Medina L., Mitschang P. (2021). Carbon Fiber Reinforced Polymers in Industrial Carbon and Graphite Materials, Volume I: Raw Materials, Production and Applications.

[10] Ma Q., Gu Y., Li M., Wang S., Zhang Z. (2016). Effects of surface treating methods of high-strength carbon fibers on interfacial properties of epoxy resin matrix composite. Appl. Surf. Sci..

[11] Keyte J., Pancholi K., Njuguna J. (2019). Recent Developments in Graphene Oxide/Epoxy Carbon Fiber-Reinforced Composites. Front. Mater..

[12] (1992). Scientific and Technical Aerospace Reports: Scientific and Technical Information Office, National Aeronautics and Space Administration. https://books.google.com.au/books?id=CLvZrzOmFFQC&printsec=frontcover&source=gbs_ge_summary_r&cad=0#v=onepage&q&f=false.

[13] Baley C., Gomina M., Breard J., Bourmaud A., Drapier S., Ferreira M., Le Duigou A., Liotier P.J., Ouagne P., Soulat D. (2018). Specific features of flax fibres used to manufacture composite materials. Int. J. Mater. Form..

[14] Lei Z., Li X., Qin F., Qiu W. (2014). Interfacial Micromechanics in Fibrous Composites: Design, Evaluation, and Models. Sci. World J..

[15] Wang B., Gao Y. (2015). Matrix formulation and interfacial enhancement of an aeronautical carbon fabric/epoxy composites fabricated via resin transfer molding (RTM) technique. J. Adhes. Sci. Technol..

[16] Zhandarov S., Mäder E. (2005). Characterization of fiber/matrix interface strength: Applicability of different tests, approaches and parameters. Compos. Sci. Technol..

[17] Budiman B.A., Takahashi K., Inaba K., Kishimoto K. (2016). Evaluation of interfacial strength between fiber and matrix based on cohesive zone modeling. Compos. Part A Appl. Sci. Manufactur..

[18] Li Q., Woodhead A.L., Church J.S., Naebe M. (2018). On the detection of carbon fibre storage contamination and its effect on the fibre–matrix interface. Sci. Rep..

[19] Petersen R.C., Lemons J.E., McCracken M.S. (2006). Stress-transfer micromechanics for fiber length with a photocure vinyl ester composite. Polym. Compos..

[20] Zhang Z.Q., Ward D.K., Xue Y., Zhang H.W., Horstemeyer M.F. (2011). Interfacial Characteristics of Carbon Nanotube-Polyethylene Composites Using Molecular Dynamics Simulations. ISRN Mater. Sci..

[21] Mengjin W., Lixia J., Suling L., Zhigang Q., Sainan W., Ruosi Y. (2021). Interfacial performance of high-performance fiber-reinforced composites improved by cold plasma treatment: A review. Surf. Interfaces.

[22] Dong J., Jia C., Wang M., Fang X., Wei H., Xie H., Zhang T., He J., Jiang Z., Huang Y. (2017). Improved mechanical properties of carbon fiber-reinforced epoxy composites by growing carbon black on carbon fiber surface. Compos. Sci. Technol..

[23] Lee S., Ko K., Youk J., Lim D., Jeong W. (2019). Preparation and Properties of Carbon Fiber/Carbon Nanotube Wet-Laid Composites. Polymers.

[24] Paiva M., Bernardo C., Nardin M. (2000). Mechanical, surface and interfacial characterisation of pitch and PAN-based carbon fibres. Carbon.

[25] Dvir H., Jopp J., Gottlieb M. (2006). Estimation of polymer–surface interfacial interaction strength by a contact AFM technique. J. Colloid Interface Sci..

[26] Jones C. (1991). The chemistry of carbon fibre surfaces and its effect on interfacial phenomena in fibre/epoxy composites. Compos. Sci. Technol..

[27] Kim J.-K., Mai Y.-W. (1991). High strength, high fracture toughness fibre composites with interface control—A review. Compos. Sci. Technol..

[28] Park S.-J., Kim B.-J. (2005). Roles of acidic functional groups of carbon fiber surfaces in enhancing interfacial adhesion behavior. Mater. Sci. Eng. A.

[29] Pukánszky B. (2005). Interfaces and interphases in multicomponent materials: Past, present, future. Eur. Polym. J..

[30] Sharma M., Gao S., Mäder E., Sharma H., Wei L.Y., Bijwe J. (2014). Carbon fiber surfaces and composite interphases. Compos. Sci. Technol..

[31] Chou T.-W., Gao L., Thostenson E.T., Zhang Z., Byun J.-H. (2010). An assessment of the science and technology of carbon nanotube-based fibers and composites. Compos. Sci. Technol..

[32] Hu C., Liao X., Qin Q.-H., Wang G. (2019). The fabrication and characterization of high density polyethylene composites reinforced by carbon nanotube coated carbon fibers. Compos. Part A Appl. Sci. Manuf..

[33] Kumar M., Kumar P., Bhadauria S.S. (2020). Interlaminar fracture toughness and fatigue fracture of continuous fiber-reinforced polymer composites with carbon-based nanoreinforcements: A review. Polym. Technol. Mater..

[34] Wang Z., Huang X., Xian G., Li H. (2015). Effects of surface treatment of carbon fiber: Tensile property, surface characteristics, and bonding to epoxy. Polym. Compos..

[35] Yao L., Cui H., Alderliesten R., Sun Y., Guo L. (2018). Thickness effects on fibre-bridged fatigue delamination growth in composites. Compos. Part A Appl. Sci. Manuf..

[36] Sheehan J.E. (1989). Oxidation protection for carbon fiber composites. Carbon.

[37] Tang B., Wang Y., Hu L., Lin L., Ma C., Zhang C., Lu Y., Sun K., Wu X. (2019). Preparation and properties of lightweight carbon/carbon fiber composite thermal field insulation materials for high-temperature furnace. J. Eng. Fibers Fabr..

[38] Tkachenko L.A., Shaulov A.Y., Berlin A.A. (2012). High-temperature protective coatings for carbon fibers. Inorg. Mater..

[39] Xiang Y., Chen Z.H., Cao F. (2014). High-temperature protective coatings for C/SiC composites. J. Asian Ceram. Soc..

[40] Gallyamova R., Galyshev S., Musin F., Badamshin A., Dokichev V. (2018). Investigation of Protective Coatings for Carbon Fibers by the Sol-Gel Method. Solid State Phenom..

[41] Chen Z., Xu C., Ma C., Ren W., Cheng H.-M. (2013). Lightweight and Flexible Graphene Foam Composites for High-Performance Electromagnetic Interference Shielding. Adv. Mater..

[42] Das A., Hayvaci H.T., Tiwari M.K., Bayer I.S., Erricolo D., Megaridis C.M. (2011). Superhydrophobic and conductive carbon nanofiber/PTFE composite coatings for EMI shielding. J. Colloid Interface Sci..

[43] Enríquez E., de Frutos J., Fernández J., de la Rubia M. (2014). Conductive coatings with low carbon-black content by adding carbon nanofibers. Compos. Sci. Technol..

[44] Micheli D., Pastore R., Apollo C., Marchetti M., Gradoni G., Primiani V.M., Moglie F. (2011). Broadband Electromagnetic Absorbers Using Carbon Nanostructure-Based Composites. IEEE Trans. Microw. Theory Tech..

[45] Mishra M., Singh A.P., Dhawan S.K. (2013). Expanded graphite–nanoferrite–fly ash composites for shielding of electromagnetic pollution. J. Alloys Compd..

[46] Narayanan T.N., Sunny V., Shaijumon M.M., Ajayan P.M., Anantharaman M.R. (2009). Enhanced Microwave Absorption in Nickel-Filled Multiwall Carbon Nanotubes in the S Band. Electrochem. Solid-State Lett..

[47] Pande S., Singh B.P., Mathur R.B., Dhami T.L., Saini P., Dhawan S.K. (2009). Improved Electromagnetic Interference Shielding Properties of MWCNT–PMMA Composites Using Layered Structures. Nanoscale Res. Lett..

[48] Singh A.P., Gupta B.K., Mishra M., Govind, Chandra A., Mathur R., Dhawan S. (2013). Multiwalled carbon nanotube/cement composites with exceptional electromagnetic interference shielding properties. Carbon.

[49] Singh A.P., Mishra M., Hashim D.P., Narayanan T., Hahm M.G., Kumar P., Dwivedi J., Kedawat G., Gupta A., Singh B. (2015). Probing the engineered sandwich network of vertically aligned carbon nanotube–reduced graphene oxide composites for high performance electromagnetic interference shielding applications. Carbon.

[50] Lubineau G., Rahaman A. (2012). A review of strategies for improving the degradation properties of laminated continuous-fiber/epoxy composites with carbon-based nanoreinforcements. Carbon.

[51] Chen I.-H., Wang C.-C., Chen C.-Y. (2010). Fabrication and Structural Characterization of Polyacrylonitrile and Carbon Nanofibers Containing Plasma-Modified Carbon Nanotubes by Electrospinning. J. Phys. Chem. C.

[52] Dai H. (2002). Carbon nanotubes: Opportunities and challenges. Surf. Sci..

[53] Sharma H., Agarwal D.C., Sharma M., Shukla A.K., Avasthi D.K., Vankar V.D. (2013). Tailoring of structural and electron emission properties of CNT walls and graphene layers using high-energy irradiation. J. Phys. D Appl. Phys..

[54] Sharma H., Kaushik V., Girdhar P., Singh V., Shukla A., Vankar V. (2010). Enhanced electron emission from titanium coated multiwalled carbon nanotubes. Thin Solid Films.

[55] Terrones M. (2003). Science and Technology of the Twenty-First Century: Synthesis, Properties, and Applications of Carbon Nanotubes. Annu. Rev. Mater. Res..

[56] Vavro J., Llaguno M.C., Satishkumar B.C., Luzzi D.E., Fischer J.E. (2002). Electrical and thermal properties of C60-filled single-wall carbon nanotubes. Appl. Phys. Lett..

[57] Barber A., Cohen S., Wagnera H.D. (2003). Measurement of carbon nanotube–polymer interfacial strength. Appl. Phys. Lett..

[58] Bekyarova E., Thostenson E.T., Yu A., Kim H., Gao J., Tang J., Hahn H.T., Chou T.-W., Itkis M.E., Haddon R. (2007). Multiscale Carbon Nanotube−Carbon Fiber Reinforcement for Advanced Epoxy Composites. Langmuir.

[59] Chen J., Ramasubramaniam R., Xue C., Liu H. (2005). A Versatile, Molecular Engineering Approach to Simultaneously Enhanced, Multifunctional Carbon-Nanotube-Polymer Composites. Adv. Funct. Mater..

[60] Gao L., Thostenson E.T., Zhang Z., Chou T.-W. (2009). Sensing of Damage Mechanisms in Fiber-Reinforced Composites under Cyclic Loading using Carbon Nanotubes. Adv. Funct. Mater..

[61] Li C., Thostenson E.T., Chou T.-W. (2008). Sensors and actuators based on carbon nanotubes and their composites: A review. Compos. Sci. Technol..

[62] Thostenson E.T., Ren Z., Chou T.-W. (2001). Advances in the science and technology of carbon nanotubes and their composites: A review. Compos. Sci. Technol..

[63] Wagner H.D. (2002). Nanotube-polyer adhesion: A mechanics approach. Chem. Phys. Lett..

[64] Thostenson E.T., Li W.Z., Wang D.Z., Ren Z.F., Chou T.W. (2002). Carbon nanotube/carbon fiber hybrid multiscale composites. J. Appl. Phys..

[65] Rahmanian S., Suraya A., Shazed M., Zahari R., Zainudin E. (2014). Mechanical characterization of epoxy composite with multiscale reinforcements: Carbon nanotubes and short carbon fibers. Mater. Des..

[66] Rahmanian S., Thean K., Suraya A., Shazed M., Salleh M.M., Yusoff H. (2012). Carbon and glass hierarchical fibers: Influence of carbon nanotubes on tensile, flexural and impact properties of short fiber reinforced composites. Mater. Des..

[67] Shazed M., Suraya A., Rahmanian S., Salleh M.A.M. (2014). Effect of fibre coating and geometry on the tensile properties of hybrid carbon nanotube coated carbon fibre reinforced composite. Mater. Des..

[68] Ghaemi F., Ahmadian A., Yunus R., Ismail F., Rahmanian S. (2016). Effects of Thickness and Amount of Carbon Nanofiber Coated Carbon Fiber on Improving the Mechanical Properties of Nanocomposites. Nanomaterials.

[69] Wang Y., Pillai S.K.R., Che J., Chan-Park M.B. (2017). High Interlaminar Shear Strength Enhancement of Carbon Fiber/Epoxy Composite through Fiber- and Matrix-Anchored Carbon Nanotube Networks. ACS Appl. Mater. Interfaces.

[70] Hung P.-Y., Lau K.-T., Fox B., Hameed N., Lee J.H., Hui D. (2018). Surface modification of carbon fibre using graphene–related materials for multifunctional composites. Compos. Part B Eng..

[71] Karakassides A., Ganguly A., Tsirka K., Paipetis A.S., Papakonstantinou P. (2020). Radially Grown Graphene Nanoflakes on Carbon Fibers as Reinforcing Interface for Polymer Composites. ACS Appl. Nano Mater..

[72] Kamae T., Drzal L.T. (2012). Carbon fiber/epoxy composite property enhancement through incorporation of carbon nanotubes at the fiber–matrix interphase—Part I: The development of carbon nanotube coated carbon fibers and the evaluation of their adhesion. Compos. Part A Appl. Sci. Manuf..

[73] Wu Y., Dhamodharan D., Wang Z., Wang R., Wu L. (2020). Effect of electrophoretic deposition followed by solution pre-impregnated surface modified carbon fiber-carbon nanotubes on the mechanical properties of carbon fiber reinforced polycarbonate composites. Compos. Part B Eng..

[74] De S., Fulmali A.O., Nuli K.C., Prusty R.K., Prusty B.G., Ray B.C. (2021). Improving delamination resistance of carbon fiber reinforced polymeric composite by interface engineering using carbonaceous nanofillers through electrophoretic deposition: An assessment at different in-service temperatures. J. Appl. Polym. Sci..

[75] Guo J., Zhang Q., Gao L., Zhong W., Sui G., Yang X. (2017). Significantly improved electrical and interlaminar mechanical properties of carbon fiber laminated composites by using special carbon nanotube pre-dispersion mixture. Compos. Part A Appl. Sci. Manuf..

[76] Stroh P. (2005). Black Pigments in Industrial Inorganic Pigments.

[77] Fukunaga A., Ueda S. (2000). Anodic surface oxidation for pitch-based carbon fibers and the interfacial bond strengths in epoxy matrices. Compos. Sci. Technol..

[78] Jang J., Yang H. (2000). The effect of surface treatment on the performance improvement of carbon fiber/polybenzoxazine composites. J. Mater. Sci..

[79] Xu Z., Huang Y., Zhang C., Chen G. (2007). Influence of rare earth treatment on interfacial properties of carbon fiber/epoxy composites. Mater. Sci. Eng. A.

[80] Kepple K., Sanborn G., Lacasse P., Gruenberg K., Ready W. (2008). Improved fracture toughness of carbon fiber composite functionalized with multi walled carbon nanotubes. Carbon.

[81] George M., Chae M., Bressler D.C. (2016). Composite materials with bast fibres: Structural, technical, and environmental properties. Prog. Mater. Sci..

[82] Gauthier M.M. (1995). Polymer-Matrix Composites. Engineered Materials Handbook Desk Edition.

[83] Pochiraju K., Tandon G.P. (2009). Interaction of oxidation and damage in high temperature polymeric matrix composites. Compos. Part A Appl. Sci. Manuf..

[84] Clarke J.L. (2003). Structural Design of Polymer Composites: Eurocomp Design Code and Background Document.

[85] Ellis B.R. (1992). Chemistry and Technology of Epoxy Resins.

[86] Nguyen-Tran H.-D., Hoang V.-T., Do V.-T., Chun D.-M., Yum Y.-J. (2018). Effect of Multiwalled Carbon Nanotubes on the Mechanical Properties of Carbon Fiber-Reinforced Polyamide-6/Polypropylene Composites for Lightweight Automotive Parts. Materials.

[87] Qin W., Chen C., Zhou J., Meng J. (2020). Synergistic Effects of Graphene/Carbon Nanotubes Hybrid Coating on the Interfacial and Mechanical Properties of Fiber Composites. Materials.

[88] Kwon Y.J., Kim Y., Jeon H., Cho S., Lee W., Lee J.U. (2017). Graphene/carbon nanotube hybrid as a multi-functional interfacial reinforcement for carbon fiber-reinforced composites. Compos. Part B Eng..

[89] Zakaria M.R., Akil H.M., Kudus M.H.A., Ullah F., Javed F., Nosbi N. (2019). Hybrid carbon fiber-carbon nanotubes reinforced polymer composites: A review. Compos. Part B Eng..

[90] Mirri F., Orloff N.D., Forster A., Ashkar R., Headrick R., Bengio E.A., Long C.J., Choi A., Luo Y., Walker A.R.H. (2016). Lightweight, Flexible, High-Performance Carbon Nanotube Cables Made by Scalable Flow Coating. ACS Appl. Mater. Interfaces.

[91] Sharma S., Lakkad S. (2011). Effect of CNTs growth on carbon fibers on the tensile strength of CNTs grown carbon fiber-reinforced polymer matrix composites. Compos. Part A Appl. Sci. Manuf..

[92] Hu D., Xing Y., Chen M., Gu B., Sun B., Li Q. (2017). Ultrastrong and excellent dynamic mechanical properties of carbon nanotube composites. Compos. Sci. Technol..

[93] Kaseem M., Hamad K., Ko Y.G. (2016). Fabrication and materials properties of polystyrene/carbon nanotube (PS/CNT) composites: A review. Eur. Polym. J..

[94] Rai A., Subramanian N., Chattopadhyay A. (2017). Investigation of damage mechanisms in CNT nanocomposites using multiscale analysis. Int. J. Solids Struct..

[95] Agnihotri P., Basu S., Kar K.K. (2011). Effect of carbon nanotube length and density on the properties of carbon nanotube-coated carbon fiber/polyester composites. Carbon.

[96] Gao S., Villacorta B., Ge L., Rufford T.E., Zhu Z. (2017). Effect of sonication and hydrogen peroxide oxidation of carbon nanotube modifiers on the microstructure of pitch-derived activated carbon foam discs. Carbon.

[97] Boroujeni A., Tehrani M., Nelson A., Al-Haik M. (2014). Hybrid carbon nanotube–carbon fiber composites with improved in-plane mechanical properties. Compos. Part B Eng..

[98] Chen J., Wu J., Ge H., Zhao D., Liu C., Hong X. (2016). Reduced graphene oxide deposited carbon fiber reinforced polymer composites for electromagnetic interference shielding. Compos. Part A Appl. Sci. Manuf..

[99] Gangineni P.K., Yandrapu S., Ghosh S.K., Anand A., Prusty R.K., Ray B.C. (2019). Mechanical behavior of Graphene decorated carbon fiber reinforced polymer composites: An assessment of the influence of functional groups. Compos. Part A Appl. Sci. Manuf..

[100] Xiao R., Ding M., Wang Y., Gao L., Fan R., Lu Y. (2021). Stereolithography (SLA) 3D printing of carbon fiber-graphene oxide (CF-GO) reinforced polymer lattices. Nanotechnology.

[101] Smith A.T., LaChance A.M., Zeng S., Liu B., Sun L. (2019). Synthesis, properties, and applications of graphene oxide/reduced graphene oxide and their nanocomposites. Nano Mater. Sci..

[102] Mohan V.B., Lau K.-T., Hui D., Bhattacharyya D. (2018). Graphene-based materials and their composites: A review on production, applications and product limitations. Compos. Part B Eng..

[103] Lawal A.T. (2020). Recent progress in graphene based polymer nanocomposites. Cogent Chem..

[104] He R., Chang Q., Huang X., Bo J. (2018). Improved mechanical properties of carbon fiber reinforced PTFE composites by growing graphene oxide on carbon fiber surface. Compos. Interfaces.

[105] Li F., Hua Y., Qu C.-B., Xiao H.-M., Fu S.-Y. (2016). Greatly enhanced cryogenic mechanical properties of short carbon fiber/polyethersulfone composites by graphene oxide coating. Compos. Part A Appl. Sci. Manuf..

[106] Okayasu M., Tsuchiya Y. (2019). Mechanical and fatigue properties of long carbon fiber reinforced plastics at low temperature. J. Sci. Adv. Mater. Devices.

[107] Islam M.S., Deng Y., Tong L., Faisal S.N., Roy A.K., Minett A.I. (2020). High grafting strength from chemically bonded 2D layered material onto carbon microfibres for reinforced composites and ultra-long flexible cable electronic devices. Mater. Today Commun..

[108] Zhang R., Gao B., Ma Q., Zhang J., Cui H., Liu L. (2016). Directly grafting graphene oxide onto carbon fiber and the effect on the mechanical properties of carbon fiber composites. Mater. Des..

[109] Ungár T., Gubicza J., Tichy G., Pantea C., Zerda T. (2005). Size and shape of crystallites and internal stresses in carbon blacks. Compos. Part A Appl. Sci. Manuf..

[110] Dannenberg E.M., Paquin L., Gwinnell H. (2000). Carbon Black in Kirk-Othmer Encyclopedia of Chemical Technology.

[111] Kühner G., Voll M. (2018). Manufacture of Carbon Black. Carbon Black.

[112] Park J.K., Do I.-H., Askeland P., Drzal L.T. (2008). Electrodeposition of exfoliated graphite nanoplatelets onto carbon fibers and properties of their epoxy composites. Compos. Sci. Technol..

[113] Li Y., Zhang H., Huang Z., Bilotti E., Peijs T. (2017). Graphite Nanoplatelet Modified Epoxy Resin for Carbon Fibre Reinforced Plastics with Enhanced Properties. J. Nanomater..

[114] Mokhena T.C., Mochane M.J., Sefadi J.S., Motloung S.V., Andala D.M. (2018). Thermal Conductivity of Graphite-Based Polymer Composites. Impact Therm. Conduct. Energy Technol..

[115] Kostagiannakopoulou C., Fiamegkou E., Sotiriadis G., Kostopoulos V. (2016). Thermal Conductivity of Carbon Nanoreinforced Epoxy Composites. J. Nanomater..

[116] Yu H.N., Lim J.W., Suh J.D., Lee D.G. (2011). A graphite-coated carbon fiber epoxy composite bipolar plate for polymer electrolyte membrane fuel cell. J. Power Sources.

[117] Aziz S., Rashid S.A., Rahmanian S., Salleh M.A.M. (2014). Experimental evaluation of the interfacial properties of carbon nanotube coated carbon fiber reinforced hybrid composites. Polym. Compos..

[118] Bedi H.S., Padhee S.S., Agnihotri P.K. (2016). On the nature of interface of carbon nanotube coated carbon fibers with different polymers. IOP Conf. Ser. Mater. Sci. Eng..

[119] Bedi H.S., Agnihotri P.K. (2019). Designing the interphase in carbon fiber polymer composites using carbon nanotubes. Procedia Struct. Integr..

[120] Singh B.P., Choudhary V., Singh V.N., Mathur R.B. (2013). Growth of carbon nanotube filaments on carbon fiber cloth by catalytic chemical vapor deposition. Appl. Nanosci..

[121] Zhang H., Liu Y., Kuwata M., Bilotti E., Peijs T. (2015). Improved fracture toughness and integrated damage sensing capability by spray coated CNTs on carbon fibre prepreg. Compos. Part A Appl. Sci. Manuf..

[122] Li M., Gu Y., Liu Y., Li Y., Zhang Z. (2012). Interfacial improvement of carbon fiber/epoxy composites using a simple process for depositing commercially functionalized carbon nanotubes on the fibers. Carbon.

[123] Altin Y., Yilmaz H., Unsal O.F., Bedeloglu A.C. (2020). Graphene oxide modified carbon fiber reinforced epoxy composites. J. Polym. Eng..

[124] Nagi C.S., Ogin S.L., Mohagheghian I., Crean C., Foreman A.D. (2020). Spray deposition of graphene nano-platelets for modifying interleaves in carbon fibre reinforced polymer laminates. Mater. Des..

[125] Gadakh D., Dashora P., Wadhankar P. (2019). A review paper on graphene coated fibres. Graphene.

[126] Sui X., Shi J., Yao H., Xu Z., Chen L., Li X., Ma M., Kuang L., Fu H., Deng H. (2016). Interfacial and fatigue-resistant synergetic enhancement of carbon fiber/epoxy hierarchical composites via an electrophoresis deposited carbon nanotube-toughened transition layer. Compos. Part A Appl. Sci. Manuf..

[127] Awan F.S., Subhani T. (2017). Preparation and Characterization of Carbon Nanotube Deposited Carbon Fiber Reinforced Epoxy Matrix Multiscale Composites. Adv. Nano Res..

[128] Yao S.-S., Jin F.-L., Rhee K.Y., Hui D., Park S.-J. (2018). Recent advances in carbon-fiber-reinforced thermoplastic composites: A review. Compos. Part B Eng..

[129] Gabrion X., Placet V., Trivaudey F., Boubakar L. (2016). About the thermomechanical behaviour of a carbon fibre reinforced high-temperature thermoplastic composite. Compos. Part B Eng..

[130] Zhang Y., Stringer J., Grainger R., Smith P.J., Hodzic A. (2013). Improvements in carbon fibre reinforced composites by inkjet printing of thermoplastic polymer patterns. Phys. Status Solidi (RRL) Rapid Res. Lett..

[131] Zhang J., Chevali V.S., Wang H., Wang C.-H. (2020). Current status of carbon fibre and carbon fibre composites recycling. Compos. Part B Eng..

[132] Ramaswamy K., O’Higgins R.M., Kadiyala A.K., McCarthy M.A., McCarthy C. (2020). Evaluation of grit-blasting as a pre-treatment for carbon-fibre thermoplastic composite to aluminium bonded joints tested at static and dynamic loading rates. Compos. Part B Eng..

[133] Quan D., Bologna F., Scarselli G., Ivankovic A., Murphy N. (2020). Interlaminar fracture toughness of aerospace-grade carbon fibre reinforced plastics interleaved with thermoplastic veils. Compos. Part A Appl. Sci. Manuf..

[134] Sebaey T.A., Bouhrara M., O’Dowd N. (2021). Fibre Alignment and Void Assessment in Thermoplastic Carbon Fibre Reinforced Polymers Manufactured by Automated Tape Placement. Polymers.

[135] Aziz S., Rashid S.A., Salleh M.A.M. (2013). Theoretical Prediction of CNT-CF/PP Composite Tensile Properties Using Various Numerical Modeling Methods. Full Nanotub. Carbon Nanostruct..

[136] Gogoi R., Sethi S.K., Manik G. (2020). Surface functionalization and CNT coating induced improved interfacial interactions of carbon fiber with polypropylene matrix: A molecular dynamics study. Appl. Surf. Sci..

[137] Hassanzadeh-Aghdam M.K., Ansari R., Darvizeh A. (2018). Micromechanical analysis of carbon nanotube-coated fiber-reinforced hybrid composites. Int. J. Eng. Sci..

[138] Rahimpour A., Madaeni S.S. (2010). Improvement of performance and surface properties of nano-porous polyethersulfone (PES) membrane using hydrophilic monomers as additives in the casting solution. J. Membr. Sci..

[139] Wang F.J., Li W., Xue M.S., Yao J.P., Lu J.S. (2011). BaTiO3–polyethersulfone nanocomposites with high dielectric constant and excellent thermal stability. Compos. Part B Eng..

[140] Li F., Liu Y., Qu C.-B., Xiao H.-M., Hua Y., Sui G.-X., Fu S.-Y. (2015). Enhanced mechanical properties of short carbon fiber reinforced polyethersulfone composites by graphene oxide coating. Polymers.

[141] Xu N., Lu C., Zheng T., Qiu S., Liu Y., Zhang D., Xiao D., Liu G. (2021). Enhanced mechanical properties of carbon fibre/epoxy composites via in situ coating-carbonisation of micron-sized sucrose particles on the fibre surface. Mater. Des..

[142] Eyckens D.J., Arnold C.L., Simon Ž., Gengenbach T.R., Pinson J., Wickramasingha Y.A., Henderson L.C. (2021). Covalent sizing surface modi-fication as a route to improved interfacial adhesion in carbon fibre-epoxy composites. Compos. Part A Appl. Sci. Manuf..

[143] Zhang J., Deng S., Wang Y., Ye L., Zhou L., Zhang Z. (2013). Effect of nanoparticles on interfacial properties of carbon fibre–epoxy composites. Compos. Part A Appl. Sci. Manuf..

[144] Siddiqui N.A., Khan S.U., Ma P.C., Li C.Y., Kim J.-K. (2011). Manufacturing and characterization of carbon fibre/epoxy composite prepregs containing carbon nanotubes. Compos. Part A Appl. Sci. Manuf..

[145] Monteserín C., Blanco M., Murillo N., Pérez-Márquez A., Maudes J., Gayoso J., Laza J.M., Hernáez E., Aranzabe E., Vilas J.L. (2019). Novel Antibacterial and Toughened Carbon-Fibre/Epoxy Composites by the Incorporation of TiO2 Nanoparticles Modified Electrospun Nanofibre Veils. Polymers.

[146] García-Moreno I., Caminero M., Rodríguez G.P., López-Cela J.J. (2019). Effect of Thermal Ageing on the Impact and Flexural Damage Behaviour of Carbon Fibre-Reinforced Epoxy Laminates. Polymers.

[147] Yu K., Shi Q., Dunn M.L., Wang T., Qi H.J. (2016). Carbon Fiber Reinforced Thermoset Composite with Near 100% Recyclability. Adv. Funct. Mater..

[148] Schultz J., Lavielle L., Martin C. (1987). The Role of the Interface in Carbon Fibre-Epoxy Composites. J. Adhes..

[149] Hughes J.D.H. (1991). The carbon fibre/epoxy interface—A review. Compos. Sci. Technol..

[150] Zakaria M.R., Akil H., Omar M.F., Abdul Kudus M.H., Mohd Sabri F.N.A., Abdullah M.M.A.B. (2020). Enhancement of mechanical and thermal properties of carbon fiber epoxy composite laminates reinforced with carbon nanotubes interlayer using electrospray deposition. Compos. Part C.

[151] Yu B., Jiang Z., Tang X.-Z., Yue C.Y., Yang J. (2014). Enhanced interphase between epoxy matrix and carbon fiber with carbon nanotube-modified silane coating. Compos. Sci. Technol..

[152] Lee G., Ko K.D., Yu Y.C., Lee J., Yu W.-R., Youk J.H. (2015). A facile method for preparing CNT-grafted carbon fibers and improved tensile strength of their composites. Compos. Part A Appl. Sci. Manuf..

[153] Yao H., Sui X., Zhao Z., Xu Z., Chen L., Deng H., Liu Y., Qian X. (2015). Optimization of interfacial microstructure and mechanical properties of carbon fiber/epoxy composites via carbon nanotube sizing. Appl. Surf. Sci..

[154] Jiang J., Xu C., Su Y., Guo Q., Liu F., Deng C., Yao X., Zhou L. (2016). Influence of Carbon Nanotube Coatings on Carbon Fiber by Ultrasonically Assisted Electrophoretic Deposition on Its Composite Interfacial Property. Polymers.

